# A Case of Acute Disseminated Encephalomyelitis in a Middle-Aged Adult

**DOI:** 10.1155/2015/601706

**Published:** 2015-06-09

**Authors:** Nicole Mahdi, Peter A. Abdelmalik, Mark Curtis, Barak Bar

**Affiliations:** ^1^Department of Neurology, Thomas Jefferson University Hospital, Philadelphia, PA 19107, USA; ^2^Department of Pathology, Thomas Jefferson University Hospital, Philadelphia, PA 19107, USA; ^3^Department of Neurosurgery, Thomas Jefferson University Hospital, Philadelphia, PA 19107, USA

## Abstract

*Objectives*. Acute disseminated encephalomyelitis (ADEM) is an inflammatory demyelinating disorder that is often preceded by infection or recent vaccination. Encephalopathy and focal neurological deficits are usually manifest several weeks after a prodromal illness with rapidly progressive neurologic decline. ADEM is most commonly seen in children and young adults, in which prognosis is favorable, but very few cases have been reported of older adults with ADEM and thus their clinical course is unknown. *Methods*. Here we present a case of ADEM in a middle-aged adult that recovered well after treatment. *Results*. A 62-year-old man presented with encephalopathy and rapid neurological decline following a gastrointestinal illness. A brain MRI revealed extensive supratentorial white matter hyperintensities consistent with ADEM and thus he was started on high dose intravenous methylprednisolone. He underwent a brain biopsy showing widespread white matter inflammation secondary to demyelination. At discharge, his neurological exam had significantly improved with continued steroid treatment and four months later, he was able to perform his ADLs. *Conclusions*. This case of ADEM in a middle-aged adult represents an excellent response to high dose steroid treatment with a remarkable neurological recovery. Thus it behooves one to treat suspected cases of ADEM in an adult patient aggressively, as outcome can be favorable.

## 1. Introduction

Acute disseminated encephalomyelitis (ADEM) is a monophasic inflammatory demyelinating disorder of the white matter that is often preceded by viral infection or recent vaccination. Encephalopathy and focal neurological deficits are usually manifest one to three weeks after a prodromal illness with neurologic decline progressing rapidly over days to weeks. Approximately 25% of patients will develop multiple sclerosis (MS) within five years of initial presentation of ADEM but the majority of individuals do not progress beyond three months [1]. ADEM is most commonly seen in children and young adults, where prognosis is favorable, but very few cases have been reported of middle-aged or elderly patients. The clinical course of these patients as compared to younger patients with ADEM is unclear. Here we present a case of ADEM in a middle-aged adult that recovered well after treatment with high dose corticosteroids.

## 2. Case Presentation

**Figure 1:**
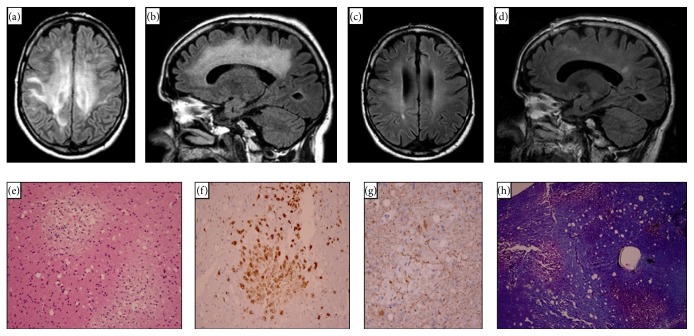
(a) Axial (T2/FLAIR) MRI showing supratentorial white matter (WM) hyperintensities extending from the periventricular region to the subcortical fibers. (b) Sagittal (T2/FLAIR) MRI showing diffuse periventricular WM intensities. (c) Axial (T2/FLAIR) MRI showing interval improvement and resolution of the periventricular WM lesions. (d) Sagittal (T2/FLAIR) MRI demonstrating the improvement of the periventricular WM lesions. (e) H&E stain shows areas of pallor containing macrophage that represent areas of demyelination (original magnification 200x). (f) Clusters of macrophages in the areas of demyelination are highlighted by immunohistochemical staining for macrophage marker CD68 (original magnification 200x). (g) Relative preservation of axons that are seen to traverse the macrophage rich areas of demyelination is demonstrated on neurofilament immunohistochemical stain (original magnification 400x). (h) Multiple pink areas of myelin loss are present in the background of normal appearing blue staining cerebral white matter on Luxol Fast Blue/PAS stain (original magnification 100x).

A 62-year-old man with a history of hypertension initially presented with progressive development of gait dysfunction, urinary incontinence, and encephalopathy over the course of two weeks following four days of a gastrointestinal illness. Upon presentation to an outside hospital, he was unable to speak and due to his depressed mental status required intubation. He underwent an MRI of the brain on the first hospital day (HD) that revealed extensive supratentorial white matter hyperintensities extending from the periventricular region to subcortical fibers without contrast enhancement (see Figures 1(a) and 1(b)). Thus he was started on 250 milligrams (mg) of intravenous (IV) methylprednisolone every 6 hours for presumed ADEM. He also underwent a lumbar puncture on HD #1 prior to administration of IV steroids that revealed an opening pressure of 20, with cerebrospinal fluid (CSF) containing 0 red blood cells, 47 white blood cells of which 85% were lymphocytes, glucose of 52, and protein of 114. He was empirically treated with acyclovir and ceftriaxone for a total of 5 days, which were discontinued after CSF bacterial cultures were negative. Blood cultures were also negative and a transthoracic echocardiogram was negative for evidence of vegetation to suggest endocarditis. Various viral titers including herpes simplex virus (HSV), Epstein-Barr virus (EBV), varicella zoster virus (VZV), and cytomegalovirus (CMV) were negative, as were serologies for CSF Lyme and* Cryptococcus neoformans*. In addition, CSF oligoclonal bands and myelin basic protein were negative. Serum inflammatory markers including ANA, C reactive protein, and sedimentation rate were not elevated. EEG showed diffuse slowing suggestive of moderate diffuse cerebral dysfunction without evidence of seizures or epileptiform activity.

Despite treatment with high dose steroids for approximately 6 days at the outside hospital, his mental status did not improve and thus he was transferred to our facility to the neurological intensive care unit for further management. Upon arrival, the patient's GCS was 10T, with spontaneous eye opening, ability to localize to pain in the left upper extremity, and intact brain stem reflexes. Additionally, he displayed triple flexion in his bilateral lower extremities and decorticate posturing in his right upper extremity. A repeat lumbar puncture performed on HD #8 demonstrated a normal opening pressure, with CSF containing 0 red blood cells, 9 white blood cells of which 94% were lymphocytes, glucose of 79, and protein of 100. CSF bacterial cultures were negative, along with no evidence of active HSV or EBV infection. CSF cytology revealed the presence of white blood cells but was negative for malignancy. RPR was also negative. Two subsequent MRIs of the brain were performed on HD #13 and HD #21 showing no significant change in the appearance of the prior white matter lesions and no new contrast enhancing lesions. In addition, an MRI of the cervical and thoracic spine with contrast performed on HD #22 did not show any prior or new enhancing white matter lesions. With continued treatment with high dose IV steroids at 250 mg of IV methylprednisolone every 6 hours for another 7 days, his mental status improved and thus he was safely extubated on HD #10 and continued on a taper of oral prednisone. He underwent a brain biopsy on HD #13 that revealed multiple small foci of macrophage accumulation and widespread white matter inflammation secondary to demyelination (see Figures 1(e) through 1(h)). He was subsequently discharged on HD #63 to acute rehab on a prolonged oral steroid taper. His physical exam on discharge was notable for a GCS of 15, fully oriented, full strength on the left, and a residual right hemiparesis. Upon outpatient follow-up four months later, he made further improvements, now ambulating without assistance, cognitively back to his baseline, and independent in his activities of daily living, with a modified Rankin score (mRS) of 0. He underwent a follow-up brain MRI five months later that showed overall improvement in the diffuse white matter changes previously seen on initial presentation (see Figures 1(c) and 1(d)).

## 3. Discussion

ADEM is theorized to be an immunologically mediated demyelinating disease triggered by a febrile illness or recent vaccination, eliciting an inflammatory response affecting the central nervous system (CNS). Possible mechanisms may include either molecular mimicry or direct inflammatory damage to myelinated neurons [2]. The prevalence of ADEM is higher in children and young adults and is thought to be related to the increased frequency of viral infections and vaccination in this patient population [3]. Radiologically, the T2/FLAIR (MRI) lesions of ADEM are diffuse, ill-defined, symmetric, often irregular, and occasionally patchy areas of homogeneous signal hyperintensities often involving both the gray and white matter of the brain with over half of cases involving infratentorial structures and greater than a third involving the spinal cord [1, 2, 4, 5]. The demonstration of multifocal hyperintensities most often affecting the subcortical white matter makes the identification of ADEM difficult given various other inflammatory, infectious, and rheumatologic disorders that have similar clinical and radiological presentations. Given such ambiguity, the International Pediatric Multiple Sclerosis Study Group proposed a consensus definition for the pediatric population with the mandatory inclusion of encephalopathy in the criteria for diagnosis [2]. Despite this useful aid in identifying young patients, there still remains no clear diagnostic criterion for ADEM in adults, and therefore older individuals are more difficult to prognosticate given the paucity of reported cases and outcomes following standard ADEM therapies such as high dose corticosteroids, intravenous immunoglobulin (IVIG), and plasmapheresis (PLEX). Even though there are no randomized control trials dictating the most effective treatment regimens for ADEM, it is frequently suggested that high dose methylprednisolone administered early in the disease course should be used as the first line therapy, since up to 80% of patients can experience full recovery [3]. More advanced therapies such as IVIG and PLEX are usually reserved for refractory or more fulminant cases.

Our patient presented with a suggestive clinical history of symptoms, physical exam, and radiological signs, along with a CSF profile and histopathology suggestive of ADEM. Given both the therapeutic and prognostic implications of considering this a case as the first demyelinating event of MS, it is important to make it clear that this most likely does not represent MS given the initial subacute presentation of encephalopathy, the presence of diffuse, multifocal, ill-defined lesions of variable morphology, and pathology showing widespread inflammation. In contrast, MS often presents with distinct episodes of focal neurological deficits with concordant confluent demyelinating lesions of varying ages appearing ovoid in shape, often affecting the white matter more than gray matter, the latter of which is seen more commonly in ADEM [1]. CSF studies reveal the persistent presence of oligoclonal bands more frequently in MS in comparison to ADEM [6]. Lesions in ADEM may also display gadolinium enhancement similar to MS but incidence can be variable or even absent depending on the time of imaging in relation to development of symptoms. It has been found that symptoms may precede MRI findings by many weeks [1, 2, 7, 8]. Given the fact that a fourth of patients with ADEM will develop MS, it is recommended that one obtains repeat imaging at three and six months to screen for the development of new lesions [1]. Furthermore in this patient, subsequent MRIs confirmed no evidence of new contrast enhancing lesions, with follow-up MRI five months later showing resolution of a monophasic demyelinating event with concomitant clinical resolution of neurological deficits. One study by O'Riordan et al. showed that ten out of eleven patients with a postinfectious encephalomyelitis had partial to complete resolution of lesions on greater than 3-month follow-up MRI, supporting the theory that ADEM lesions regress on MRI, treatment of which is not typically seen in MS [4, 5].

Relapsing-remitting MS (RRMS) remains the most common form of idiopathic inflammatory demyelinating disease (IIDD) but other fulminant IIDDs such as Marburg disease (MD), Schilder's disease (SD), and Balo's concentric sclerosis (BCS) should also be considered when evaluating patients with atypical clinical and radiological courses not consistent with classic RRMS. MD, similar to ADEM, is characterized by confusion, headache, and focal deficits with multiple often coalescing focal enhancing lesions on T2/FLAIR MRI but has a rapidly progressive course with relapses and high rate of morbidity and mortality within weeks to months of onset [1, 9]. SD and BCS are less often confused with typical ADEM given the more rapid progression to morbidity and the distinct radiological findings of large, symmetric, incompletely ring-enhancing white matter lesions in SD and alternating layers of demyelination amongst rims of preserved myelin that resemble “onion bulbs” on MRI [1, 9]. Additionally, this patient did not display any clinical or radiological signs of a myelopathy or radiculopathy that would suggest a combined demyelinating syndrome involving both the peripheral and central nervous systems, which has been described in a recent Italian study looking at the heterogeneity of postinfectious neurological syndromes [10].

A small case series of middle-aged adults presenting with ADEM has been described by Wang et al., in which all three patients had a single episode of focal neurological deficits and encephalopathy with typical MRI findings and clinically improved with steroid treatment [11]. A larger cohort of forty patients with ADEM were observed by Schwarz et al. to determine the possibility of both identifying and prognosticating those patients who eventually develop clinical and radiological MS. Of those twenty-six patients given a final diagnosis of ADEM, which included ages ranging from 19 to 61, a more favorable response to therapy was observed despite more severe initial symptoms than those who met criteria for a diagnosis of MS [8]. Although typically reserved for more fulminant forms of ADEM, there have also been individual reports of the concomitant use of steroids along with IVIG and PLEX in the treatment of ADEM in older patients with favorable outcomes, suggesting that this presentation in older adults may in fact be of a similar pathophysiology that is well described in children and young adults [12, 13].

ADEM rarely presents in the middle-aged to elderly adult and due to the paucity of cases reported in the literature, the prognosis in this age group is unknown. We present a case of ADEM in a middle-aged adult in which the patient had an excellent response to treatment with high dose steroids, resulting in a remarkable neurological recovery. Thus it behooves us to treat suspected cases of ADEM in an adult patient aggressively, as outcome may be favorable.

## Supplementary Material

Supplementary Figure 1: MRI imaging on admission [A through D] in comparison to those obtained on 7 month follow up [E & F]. [A] Axial T2/FLAIR showing supratentorial WM hyperintensities extending from the periventricular region through the subcortical fibers. [B] Axial view diffusion-weighted imaging showing no restricted diffusion to suggest acute infarction. [C] Axial T1 post-contrast image showing no contrast enhancement of the T2/FLAIR lesions. [D] Sagittal T2/FLAIR image showing diffuse periventricular WM intensities. [E] Axial T2/FLAIR showing interval improvement & resolution of the periventricular WM lesions. [F] Sagittal T2/FLAIR also demonstrating the improvement of the periventricular WM lesions.
Supplementary Figure 2: T2 weighted mid sagittal MRI images of the cervical, [A], and thoracic, [B], spinal cord demonstrating no spinal cord signal hyperintensities, although with mild spinal canal narrowing at multiple levels, most severe at C3-C4, due to cervical disk protrusion.
