# Supplementary material for: A Case of Acute Disseminated Encephalomyelitis in a Middle-Aged Adult
Source: Case Rep Neurol Med. 2015 Jun 9;2015:601706. doi: 10.1155/2015/601706 (PMC4477182; doi:10.1155/2015/601706)
Supplement: Supplementary file 1 — Supplementary Figure 1: MRI imaging on admission [A through D] in comparison to those obtained on 7 month follow up [E & F]. [A] Axial T2/FLAIR showing supratentorial WM hyperintensities extending from the periventricular region through the subcortical fibers. [B] Axial view diffusion-weighted imaging showing no restricted diffusion to suggest acute infarction. [C] Axial T1 post-contrast image showing no contrast enhancement of the T2/FLAIR lesions. [D] Sagittal T2/FLAIR image showing diffuse periventricular WM intensities. [E] Axial T2/FLAIR showing interval improvement & resolution of the periventricular WM lesions. [F] Sagittal T2/FLAIR also demonstrating the improvement of the periventricular WM lesions. Supplementary Figure 2: T2 weighted mid sagittal MRI images of the cervical, [A], and thoracic, [B], spinal cord demonstrating no spinal cord signal hyperintensities, although with mild spinal canal narrowing at multiple levels, most severe at C3-C4, due to cervical disk protrusion. [file 601706.f1.docx]

**Supplementary Figures**

**Figure 1**

**
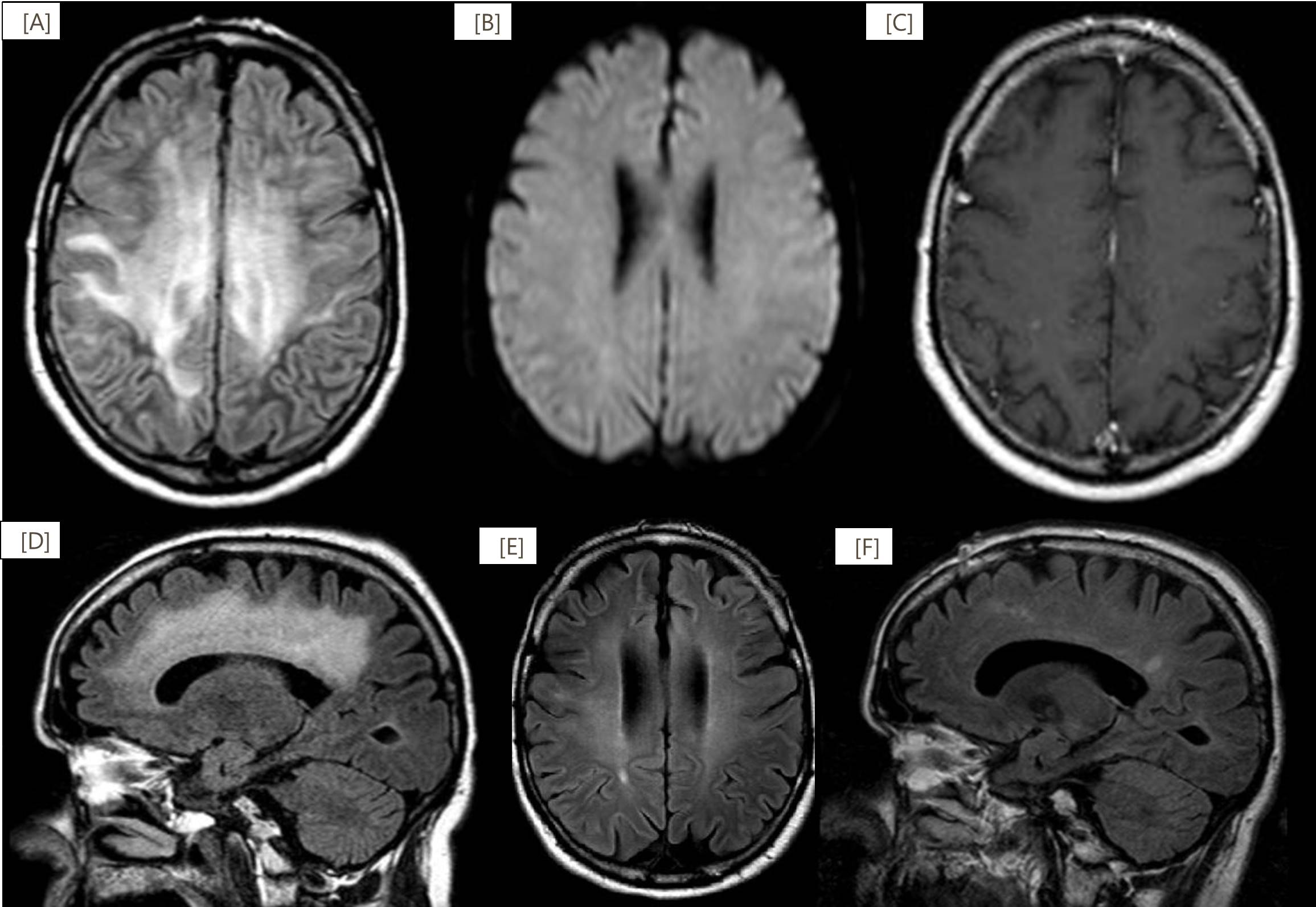
**

*Figure 1*: MRI imaging on admission [A through D] in comparison to those obtained on 7 month follow up [E & F]. [A] Axial T2/FLAIR showing supratentorial WM hyperintensities extending from the periventricular region through the subcortical fibers. [B] Axial view diffusion-weighted imaging showing no restricted diffusion to suggest acute infarction. [C] Axial T1 post-contrast image showing no contrast enhancement of the T2/FLAIR lesions. [D] Sagittal T2/FLAIR image showing diffuse periventricular WM intensities. [E] Axial T2/FLAIR showing interval improvement & resolution of the periventricular WM lesions. [F] Sagittal T2/FLAIR also demonstrating the improvement of the periventricular WM lesions.

**Figure 2**

**
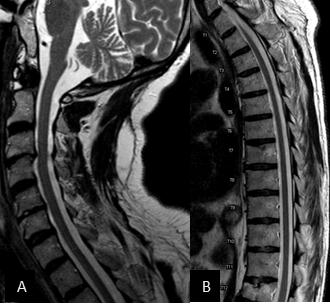
**

*Figure 2*: T2 weighted mid sagittal MRI images of the cervical, [A], and thoracic, [B], spinal cord demonstrating no spinal cord signal hyperintensities, although with mild spinal canal narrowing at multiple levels, most severe at C3-C4, due to cervical disk protrusion.
